# Climate change and extreme weather disasters: evacuation stress is associated with youths’ somatic complaints

**DOI:** 10.3389/fpsyg.2023.1196419

**Published:** 2023-06-22

**Authors:** Annette M. La Greca, Evan T. Burdette, Kaitlyn E. Brodar

**Affiliations:** Department of Psychology, College of Arts and Sciences, University of Miami, Coral Gables, FL, United States

**Keywords:** somatic complaints, adolescents, children, evacuation, climate change, disasters

## Abstract

**Objective:**

Climate-change has brought about more frequent extreme-weather events (e.g., hurricanes, floods, and wildfires) that may require families to evacuate, without knowing precisely where and when the potential disaster will strike. Recent research indicates that evacuation is stressful for families and is associated with psychological distress. Yet, little is known about the potential impact of evacuation stressors on child health. After Hurricane Irma, which led to a mass evacuation in Florida, we examined whether evacuation stressors and hurricane exposure were uniquely associated with youth somatic complaints, and whether youth psychological distress (i.e., symptoms of posttraumatic stress, anxiety, and depression) served as a potential mediating pathway between evacuation stressors, hurricane experiences, and somatic complaints.

**Method:**

Three months after Irma, 226 mothers of youth aged 7–17 years (*N*=226; *M* age = 9.76 years; 52% boys; 31% Hispanic) living in the five southernmost Florida counties reported on evacuation stressors, hurricane-related life threat and loss/disruption, and their child’s psychological distress and somatic complaints using standardized measures.

**Results:**

Structural equation modeling revealed a good model fit (χ^2^ = 32.24, *p* = 0.003, CFI = 0.96, RMSEA = 0.08, SRMR = 0.04). Even controlling for life-threatening hurricane experiences (*β* = 0.26) and hurricane loss and disruption (*β* = 0.26), greater evacuation stressors were associated with greater symptoms of youth psychological distress (*β* = 0.34; *p*’s < 0.001), and greater psychological distress was associated with more somatic complaints (*β* = 0.67; *p* < 0.001). Indirect effects revealed that evacuation stressors (*p* < 0.001), actual life-threatening events (*p* < 0.01), and loss and disruption (*p* < 0.01) were all uniquely and indirectly associated with youths’ somatic complaints via youth psychological distress.

**Discussion:**

Findings suggest that even coping with the *threat of a disaster* may be sufficient to prompt psychological and physical health symptoms in youth. Due in part to climate change, threats of disaster occur much more often than actual disaster exposure, especially for areas that are prone to hurricanes or wildfires. Preparing youth and families residing in vulnerable areas for potential disaster evacuation or sheltering-in-place appears critical. Encouraging families to develop Disaster Plans and teaching stress management skills may reduce both youth distress and somatic health problems.

## 1. Introduction

Climate-change has brought about increases in the frequency, intensity, and duration of extreme climate events such as wildfires, as well as flooding due to hurricanes, coastal storms, and extreme precipitation (Bell et al., 2016). For example, in 2020, the U.S. set a record for the most billion-dollar weather and climate disaster events in a calendar year (*N* = 22). Both 2021 and 2022 followed closely behind with 20 and 18 separate billion-dollar climate events, respectively (Smith, 2022, 2023). Similarly, in Australia and other parts of the world, the period from 2019 to 2020 witnessed many climate-related extreme weather events (i.e., fires, cyclones, floods (Rankin, 2020; Cave, 2021; Steffen, 2021)).

Such extreme events pose a serious threat to human health (Crimmins et al., 2016). For example, flooding and wildfires have been found to contribute to a variety of health hazards, such as injury, illness, and an exacerbation of underlying medical conditions (Dodgen et al., 2016). Further, research has established that climate-related disasters contribute to the development of psychological distress, and especially to symptoms of posttraumatic stress (PTS), anxiety, and depression in both youth and adults (Hardin et al., 1994; Khoury et al., 1997; Neria et al., 2008; Bonanno et al., 2010; Furr et al., 2010; La Greca et al., 2010, 2013a,b).

In particular, hurricanes are one of the most well studied climate-related disasters and are expected to become more frequent and intense in coming years (Holland and Bruyère, 2014; Bell et al., 2016; Grinsted et al., 2019). Given the significant population already residing in coastal areas, and recent population gains in disaster-prone areas such as Florida and Texas (Kamin, 2023), large segments of the U.S. population are at risk for experiencing a major hurricane, storm surge, or flood (Bathi and Das, 2016).

Importantly, even if a potentially life-threatening storm does not ultimately strike, the uncertainty around disaster forecasting (Henson, 2022) means that large segments of the population may potentially be in harm’s way and need to evacuate to ensure safety (Federal Emergency Management Association, n.d.; Centers for Disease Control and Prevention, 2022). However, little is known about the potential impact of evacuation-related stressors on youths’ physical health problems; this issue was the focus of the current study.

### 1.1. Evacuation stressors

Evacuation is among the most widely used strategies for protecting lives in the face of impending disasters such as hurricanes and wildfires (Wolshon et al., 2005a,b; McLennan et al., 2019). The decision to evacuate or not, as well as the actual process of evacuation, is reported to be stressful for families (Smith and McCarty, 2009), yet has received limited empirical attention.

Most evacuation studies focus on factors related to adults’ *intentions* to evacuate, or on social and demographic differences between those who do versus do not evacuate (e.g., Kulkarni et al., 2017). Among the available findings, it appears that adults’ perceived risk and concerns about family safety play a role in their decisions to evacuate (Edwards and Bateman, 2002; Eisenman et al., 2007; Smith and McCarty, 2009; Thompson et al., 2016; Kulkarni et al., 2017); these studies also find that women and households with children are more likely to evacuate for an impending disaster (than men or childless households), presumably due to family safety concerns.

Less attention has been devoted to detailing the aspects of the evacuation process that are stressful for families. We recently reported on families’ hurricane-related evacuation experiences (La Greca et al., 2019), finding that the evacuation process is fraught with numerous logistic problems that are stressful. Even before a storm, the most frequent and stressful events parents report include (a) *difficulties in decision-making* (e.g., deciding whether to stay or leave; disagreements with family members about whether to evacuate or not, or about what to bring versus leave behind), (b) *transportation challenges* (e.g., trouble getting gasoline for the car; getting stuck in traffic or other travel delays; car breaking down; airline flight cancelled), (c) *family separation* (e.g., separating from a key family member, typically due to work constraints), and (d) *trouble finding a place to stay or getting into a shelter*. Many of these evacuation stressors were reported for families who evacuated as well as for those who ultimately sheltered-in-place. Further, qualitative interviews with the families who did evacuate disclosed that the worst part of evacuating was “stress, worry, and uncertainty” (both in general, as well as specific worries about their home and belongings), along with stressful travel experiences.

Not surprisingly, then, families that evacuated reported significantly higher levels of stress *both before and after the storm* than did those who sheltered-in-place. On the positive side, however, despite substantial evacuation-related stress, families who evacuated reported feeling significantly safer *during* the storm, than those who sheltered-in-place (La Greca et al., 2019).

As a recent follow-up to this study, we examined whether evacuation-related stressors were associated with parents’ and youths’ psychological distress, *even when controlling for their actual disaster-related experiences* (La Greca et al., 2022). As noted above, youths’ and adults’ direct exposure to climate-related disasters is associated with psychological distress (e.g., Khoury et al., 1997; Neria et al., 2008; Bonanno et al., 2010; Furr et al., 2010; La Greca et al., 2010, 2013a,b). Our findings revealed that evacuation stressors were uniquely associated with parents’ and youths’ psychological distress (as assessed by symptoms of PTS, anxiety, and depression) even when controlling for hurricane experiences, such as life threat during the storm and loss/disruption after the storm.

The present study extended the above findings by evaluating whether “before the storm” evacuation stressors were also associated with youths’ health problems; specifically, with youths’ somatic complaints. We examined this issue while controlling for youths’ direct hurricane-related exposure, and also evaluated whether psychological distress served as a potential mediator between evacuation stressors and youths’ health problems.

### 1.2. Natural disasters and child health

The impact of natural disasters on child health represents a major gap in the traumatic stress literature (La Greca et al., 2016). This is surprising, in that stress has long been viewed as playing a role in health and illness (e.g., Seiler et al., 2020; American Psychological Association, 2023). Moreover, the life disruption associated with disasters can interfere with getting adequate sleep and exercise (e.g., Brown et al., 2011; Lai et al., 2013, 2020), which can also affect one’s health in the short- and long-term.

With respect to hurricanes, among the scant findings, Hensley and Varela (2008) surveyed middle school youth (average age of 12 years) in the New Orleans area 5–8 months after Hurricane Katrina. They assessed youths’ hurricane exposure, symptoms of PTS, trait anxiety, and somatic complaints. The most commonly reported somatic complaints were headaches and nausea/upset stomach. Age, ethnic minority status, and family income were unrelated to youths’ somatic complaints, although girls reported more somatic complaints than boys. These authors found that hurricane exposure was a significant predictor of youths’ somatic symptoms.

Subsequently, Lai et al. (2014) examined the association between disaster exposure, other life stressors, and sedentary behavior in youth (average age 9 years) 8 months after Hurricane Ike struck Galveston, Texas. Findings revealed that hurricane exposure (i.e., life threat, loss and disruption), as well as major stressors that occurred during recovery period, were significantly associated with youths’ symptoms of PTS, which in turn were associated with increased time spent in sedentary activities.

Finally, Felix et al. (2016) investigated the long-term association between disaster exposure and youths’ physical health. Parents of youth (aged 6–17 years) were interviewed 18 and 30 months after Hurricane Georges struck Puerto Rico and reported on their child’s hurricane exposure along with several aspects of their child’s physical health (e.g., global health, number of medical problems, and number of medical visits). At both timepoints, greater hurricane exposure was significantly associated with poorer overall youth health and a greater number of medical visits and medical problems.

Overall, the above studies suggest that disasters have the potential to adversely affect youths’ long-term physical health. The current study replicated and significantly extended the above findings in two ways. First, we examined whether hurricane-related experiences of life-threat and loss/disruption were associated with reports of somatic complaints (e.g., stomachaches, headaches, nausea, and colds) in a broad age-range of youth (7 to 17 years). We focused on somatic complaints as our primary health outcome because such symptoms are associated with poor school attendance (e.g., Bernstein et al., 1997; Havik et al., 2015; Li et al., 2021), greater functional disability (e.g., Cerutti et al., 2017), and increased medical care usage (e.g., Campo et al., 1999; Heimann et al., 2018; Saunders et al., 2020). Second, we evaluated whether evacuation stressors uniquely contributed to youths’ somatic symptoms, controlling for disaster exposure. To our knowledge, no prior studies have evaluated whether evacuation-related stressors contribute to youths’ post-disaster health problems.

### 1.3. Psychological distress as a potential mediator of the association between evacuation stressors and youth health

Evacuation stressors could potentially affect youths’ somatic symptoms directly, given the well-documented association between stress and physical health (Caserta et al., 2008; Kalmakis and Chandler, 2015). However, it is also possible that youths’ psychological distress could serve as a mediating pathway between evacuation stressors and youths’ somatic complaints.

For example, a study by Lai et al. (2020) after Hurricane Ike found that sleep problems over time were best predicted by children’s reports of hurricane-related PTS rather than by hurricane exposure (Lai et al., 2020). Another study conducted after Hurricane Ike (and described above), found that children’s symptoms of PTS mediated the association between hurricane exposure and youths’ sedentary behavior (Lai et al., 2014). Sedentary behavior, in turn, has been recognized as a risk factor for poor health (e.g., Tremblay et al., 2011).

Accordingly, in the current study, we examined whether evacuation stressors and hurricane exposure were directly associated with youths’ somatic complaints or were indirectly associated via their linkages with psychological distress. Identifying potential mediating pathways between evacuation stressors and health is important to better understand how to address and prevent evacuation stressors from having an adverse effect on youth.

### 1.4. The current study

Hurricane Irma provided an opportunity to evaluate the association between evacuation-related stressors and youths’ somatic complaints. This focus is important in that youth have been identified as a vulnerable population in disasters (Norris et al., 2002; Bonanno et al., 2010; Felix et al., 2020). Specifically, we evaluated whether evacuation-related stressors were associated with indicators of youths’ somatic complaints (e.g., headaches, stomachaches, and colds/flu), even after accounting for their actual exposure to Hurricane Irma (i.e., life-threatening events during the storm, loss and disruption after the storm).

Hurricane Irma was significant in that it led to one of the largest mass evacuations in U.S. history, with nearly 7 million Florida residents evacuating their homes for safety reasons (Allen, 2018). This mass evacuation was precipitated by weather forecasts indicating that Irma was one of the strongest hurricanes ever recorded in the Atlantic basin and was projected to hit Southern Florida as a Category 5 hurricane, with winds exceeding 150 miles per hour, before heading up through the state (National Weather Service, n.d.; Chokshi and Haag, 2017). Although Irma eventually made landfall in the Florida Keys and Southwest Florida as a Category 4 and 3 hurricane, respectively, the entire southern end of the Florida peninsula was affected (NOAA National Centers for Environmental Information, 2018).

To examine whether pre-disaster evacuation stressors and actual hurricane exposure were uniquely associated with youths’ somatic complaints, we built on a conceptual model of risk and resilience that has been useful in understanding factors associated with adverse post-disaster outcomes (Bonanno et al., 2010; La Greca et al., 2010). The full “risk and resilience” model includes (a) *individual pre-disaster characteristics* (e.g., gender, ethnicity, socioeconomic status), (b) *disaster exposure* (e.g., life-threat during the event, post-disaster loss and disruption), and (c) *recovery variables* (e.g., availability of social support) as predictors of distress (La Greca et al., 1996, 2010). The current study focused on the first two factors in the model.

Specifically, with respect to pre-disaster characteristics, we examined whether youths’ gender, ethnicity, and socioeconomic status were associated with youths’ somatic complaints. Prior research suggests that girls and youth from low-income or ethnic minority backgrounds may be more vulnerable to disasters (e.g., Hensley and Varela, 2008; Hallegatte et al., 2020; Arlinghaus et al., 2023). With respect to disaster exposure, we evaluated the associations between hurricane-related life threat, loss and disruption, and youths’ somatic complaints. Moreover, we extended the risk-and-resilience model by evaluating evacuation stressors as a unique aspect of “disaster exposure;” that is, we examined whether evacuation stressors contributed to youths’ somatic complaints above and beyond the expected impact from hurricane-related life-threat and loss/disruption. Finally, we evaluated the possibility that evacuation stressors and hurricane experiences were associated with youths’ somatic problems directly or primarily *via* their linkages with psychological distress.

## 2. Methods and measures

### 2.1. Participants

Participants were 226 mothers of youth aged 7–17 years; youth functioning was obtained from parental reports. Table 1 summarizes participant characteristics. Most mothers were White (89%) and over 35 years of age (61%); 27% reported their ethnicity as Hispanic/Latinx; less than half the mothers (40%) completed a college degree; and 37% reported low family income (under $50,000 per year). On average, the youth were approximately 10 years of age (*M* age = 9.76 years, SD = 2.55); about half (51%) were boys and 31% were from Hispanic/Latinx backgrounds.

**Table 1:** Socio-demographic characteristics of the parents (*N* = 226) and youth (*N* = 226).

Characteristic	Mothers *N* (%)	Youth *N* (%)
Gender		
Female	226 (100%)	110 (48.67%)
Age		
Mother under 35 years	89 (39.4%)	–
Youth (in years)	–	*M* = 9.76 (SD = 2.55)
Ethnic/racial minority	81 (35.84%)	102 (45.13%)
Hispanic (yes)	62 (27.43%)	70 (31.00%)
White	201 (88.94%)	184 (81.42%)
Black	13 (5.75%)	17 (7.52%)
Asian	3 (1.33%)	1 (0.44%)
Multi-racial/other	9 (3.98%)	24 (20.62%)
Mother’s highest level of education		
High school or less	36 (15.93%)	–
Some college/associate’s	99 (43.81%)	–
Bachelor’s degree	56 (24.78%)	–
Graduate degree	35 (15.49%)	–
Family gross annual income		
Less than $50,000	83 (36.73%)	–
$50,000–$75,000	64 (28.32%)	–
$75,000–$100,000	34 (15.04%)	–
More than $100,000	45 (19.91%)	–
Number of children in the home	*M* = 2.47 (SD = 1.09)	*M* = 2.47 (SD = 1.09)
Lived in mandatory evacuation zone (yes)	115 (50.89%)	115 (50.89%)
Evacuated for Irma (yes)	128 (56.64%)	128 (56.64%)

Initial inclusion criteria for this study were: (a) being a parent aged 18 years or older, (b) having a child under 18 years living at home, (c) having the ability to read English or Spanish, and (d) residing in one of the five southernmost Florida (FL) counties that were affected by Hurricane Irma (Lee, Collier, Broward, Miami-Dade, and Monroe). Mothers reported on one of their children living at home. In terms of exclusion criteria, because most respondents initially were mothers (97%), we limited the sample to mothers; and because the measures of youth mental and physical health were valid for youth aged 7–17 years, only mothers’ reports of youth in this age range (*N* = 226) were included.

According to the 2020 Census Data (US Census Bureau, 2023), mothers in our sample were broadly representative of adults from the five targeted FL counties, except for a somewhat higher proportion of White adults and lower proportion of Black adults (5.8%). The proportion of mothers from Hispanic/Latinx backgrounds was similar to that of individuals in the five southern FL counties (range of 22%–31%), except for Miami-Dade which has a higher percentage of Hispanic/Latinx individuals (69%). In this sample, about half the mothers (50.9%) reported living in a mandatory evacuation zone, and 76.5% of these families evacuated for Irma. Even among those not residing in a mandatory evacuation zone (49.1%), 36% reported evacuating. Families in evacuation zones were significantly more likely to evacuate than those not residing in mandatory zones (χ^2^ = 37.70, *p* < 0.001).

### 2.2. Procedures

This study was part of a larger project on the impact of evacuation stressors on families. The relevant Institutional Review Boards reviewed and approved the study procedures.

Parents were recruited to participate and report on their child’s functioning using boosted Facebook ads. This method of recruitment was chosen because of the high prevalence of Facebook use among adults from diverse ethnic and racial backgrounds (Perrin and Anderson, 2019). Parents were informed that the study was about the experiences of families before, during, and after Hurricane Irma. Using a secure online platform, parents provided consent and completed questionnaires anonymously. Consent forms and questionnaires were provided in both English and Spanish, although nearly all participants (99%) completed the forms in English. The questionnaires were completed 3–4 months after Irma (i.e., mid-December 2017 to mid-January 2018). Mothers reported on demographics, evacuation stressors, hurricane-related experiences during and after the storm, and their youth’s current psychological distress and somatic health problems.

We followed a standard protocol to determine the validity of the parental report (e.g., checking that the participant’s stated home address was valid and located in a target county; having several “roadblock” items to ensure responses were from an attentive human; and checking for response consistency). Compensation was provided in the form of a $25 gift card, or mothers could choose to donate their $25 to a hurricane relief fund.

### 2.3. Measures

Mothers provided information about their age, race/ethnicity, and family income and also reported their child’s age, gender, and race/ethnicity. Mothers then completed the following measures for their child.

#### 2.3.1. Evacuation-stressors

Evacuation-related stressors were assessed by *Before and After the Storm Experiences (BASE)* (La Greca et al., 2019). The 21 BASE items reflect objective experiences related to evacuation (e.g., *Trouble making decisions about whether to stay or leave, getting stuck in a lot of traffic*) occurring before and after the storm. Parents rated each item for stressfulness (1 = *did not happen/was not stressful* to 4 = *very stressful*). We used the “before the storm” subscale (15 items; Cronbach’s alpha = 0.83) in this study, as prior work indicated that “before the storm” scores were significantly related to perceptions of stress before a storm (La Greca et al., 2019).

#### 2.3.2. Hurricane exposure

Items from *the Hurricane-Related Traumatic Experiences-II (HURTE-II;* La Greca, 2017) assessed youths’ exposure to Hurricane Irma. The HURTE is a widely-used measure to assess hurricane exposure (e.g., Hensley and Varela, 2008; La Greca et al., 2010, 2013a,b; Lai et al., 2013, 2014; Felix et al., 2016). Mothers completed the HURTE-II for their child; all mothers reported being with their child during the hurricane. Items were worded to reflect objective experiences that occurred during or after the hurricane. Specifically, items pertained to (a) actual life-threatening events during the storm (e.g., *Did windows or doors break in the place your child was staying?* 11 items, each rated Yes/No and summed), and (b) loss/disruption events occurring after the storm (e.g., *Did your child have to go to a new school because of the hurricane?* 14 items; each rated Yes/No and summed). Hurricane-exposure measures of actual life-threat and loss/disruption have been widely used and are predictive of youths’ psychological distress after disasters (e.g., La Greca et al., 2010, 2013a,b; Lai et al., 2013, 2014; Paul et al., 2014). As in prior research (e.g., Hensley and Varela, 2008; La Greca et al., 2010; Lai et al., 2013; Felix et al., 2016) internal consistency was not calculated, as the events that comprise these measures are diverse and may or may not co-occur.

#### 2.3.3. Youth psychological distress

Three measures assessed youths’ psychological distress for the past month. First, youth’s PTS was assessed using the well-validated *UCLA PTSD Reaction Index-V* (*PTSD-RI-V*; Kaplow et al., 2020). It contains 27 items (e.g., *My child tries to stay away from people, places, or things that remind him/her about the hurricane*), rated from 0 (*none*) to 4 (*most of the time*) and summed. In this study, internal consistency was *α* = 0.95.

Second, youths’ symptoms of anxiety were assessed with the Generalized Anxiety Disorder subscale of the *Screen for Child Anxiety Related Emotional Disorders* (*SCARED;* Birmaher et al., 1997). This subscale contains nine items (e.g., *my child is a worrier*) that are rated from 0 (*not true or hardly ever true*) to 2 (*very true or often true*) and summed. The parent-version of the *SCARED* demonstrates good internal consistency and discriminant validity (Birmaher et al., 1997). In this study, internal consistency was *α* = 0.93.

Finally, youths’ depressive symptoms were assessed using the parent-report version of the *Revised Child Anxiety and Depression Scale* (RCADS; Chorpita et al., 2005). The *RCADS* depression subscale contains 10 items (e.g., *my child feels sad or empty*) that are rated from 0 (*never*) to 3 (*always*) and summed. The subscale has good convergent and discriminant validity, as well good internal consistency (*α* = 0.87; Chorpita et al., 2005). In this study, internal consistency was *α* = 0.88.

#### 2.3.4. Somatic complaints

Youths’ somatic complaints over the past month were assessed with 7 items from the Somatic Subscale of the *Child Behavior Checklist* (Achenbach et al., 1991, 2003). Items included aches or pains (not due to stomachaches or headaches), headaches, nausea, stomachaches, vomiting, rashes or other skin problems, and colds/flu. Items that assessed anxiety-related symptoms (e.g., nightmares) were excluded. Items were scored 1 for “Not true,” 2 for “Somewhat or sometimes true,” and 3 for “Very true or often true.” In this study, internal consistency was *α* = 0.79.

### 2.4. Data analysis

Using SPSS Version 27, descriptive statistics were calculated for demographic variables. Next, missingness was analyzed. For youth, 10.5% was missing, primarily due to missingness for the HURTE-II “during the storm” items. The main analyses used full information maximum likelihood (FIML) estimation with MPLUS Version 8.8 (Muthen and Muthen, 2017). FIML estimation was used to obtain descriptive statistics for evacuation stressors, hurricane exposure, psychological distress, and somatic complaints, as well as correlations among these variables.

Primary analyses used structural equation modeling (SEM) to evaluate the associations between evacuation stressors, hurricane exposure, and youths’ somatic complaints and psychological distress. SEM was used to estimate a youth psychological distress latent variable with PTS and symptoms of general anxiety and depression as indicators. Then, to evaluate direct effects, youths’ somatic complaints and psychological distress were regressed on gender, family income, evacuation stressors, and hurricane exposure. Gender was included as a covariate as gender differences are commonly observed in psychological distress and somatic complaints (e.g., Hankin et al., 1998; Hensley and Varela, 2008). Family income also was included as a covariate as it was (negatively) related to evacuation stressors and hurricane-related loss/disruption. However, because race and ethnicity were unrelated to key study variables these variables were not included in SEM analyses.

Finally, to test for potential mediating pathways, we analyzed the indirect effects of evacuation stressors, life-threatening events during the storm, and loss/disruption after the storm on youth somatic problems *via* psychological distress. Good model fit for the structural model was determined by examining the chi-square test of significance, RMSEA < 0.06, CFI > 0.95, and SRMR < 0.08 cutoffs based on guidelines described by West et al. (2012).

## 3. Results

### 3.1. Preliminary analyses

Table 2 presents the means and standard deviations for the key study variables. In terms of evacuation stressors, mothers reported that their child experienced moderate levels of evacuation-stressors before the storm. Mothers reported that, on average, their child experienced relatively low levels of hurricane exposure, with about one actual life-threatening event during the storm (e.g., doors or windows breaking in the home) and less than one major loss and disruption event after the storm (e.g., family had to move). On average, the mean values for youth PTS (*M* = 5.95; SD = 10.73), anxiety (*M* = 4.03; SD = 4.42), and depression (*M* = 1.80; SD = 3.12) all fell within the non-clinical range. However, 5.5% of the youth exceeded clinical cutoffs for PTS, 1.8% exceeded cutoffs for anxiety, and 15.8% exceeded cutoffs for depression. On average, youth were reported to have mild to moderate levels of somatic complaints (about 2 of 7 symptoms were endorsed; *M* = 8.88; SD = 2.32) 3–4 months after the storm. The most commonly endorsed somatic symptoms were colds/flu, headaches, and stomachaches. No significant differences were found in these key study variables based on youths’ gender or ethnicity (*p*’s > 0.05).

**Table 2:** Means for evacuation stressors, hurricane exposure, psychological distress and somatic complaints.

Variable	Mean	SD	Minimum	Maximum	% Meeting clinical cutoff
Evacuation stressors	28.09	8.29	15.00	59.00	–
Hurricane-exposure					
Life threat during	0.79	0.79	0.00	3.00	–
Loss and disruption after	0.43	0.88	0.00	5.00	–
Posttraumatic stress	5.95	10.73	0.00	59.00	5.5
Anxiety	4.03	4.42	0.00	18.00	1.8
Depression	1.80	3.12	0.00	20.00	15.8
Somatic complaints	8.88	2.32	7.00	19.00	–

Table 3 contains bivariate correlations among study variables. Moderate to high associations were observed among the indicators of psychological distress (*r*’s from 0.60 to 0.74). Associations among evacuation and hurricane exposure variables were generally low (*r*’s from 0.08 to 0.22). Both hurricane exposure variables were associated with youth somatic complaints (*r*’s 0.17 and 0.14, *p*’s < 0.05). Youth somatic complaints were associated with youths’ PTS (*r* = 0.50, *p* < 0.001), anxiety symptoms (*r* = 0.46, *p* < 0.001), and depressive symptoms (*r* = 0.57, *p* < 0.001). Family income was negatively correlated with evacuation stressors (*r* = −0.13, *p* = 0.05) and loss/disruption (*r* = −0.19, *p* < 0.01), indicating that those with lower income reported more evacuation stressors and more loss/disruption after the storm. Lower income was also associated with greater youth PTS (*r* = −0.18, *p* < 0.01) and depressive symptoms (*r* = −0.18, *p* < 0.01) following the storm.

**Table 3:** Correlations for evacuation stressors, hurricane exposure, psychological distress, and somatic complaints (*N* = 226).

	1	2	3	4	5	6	7
1. Evacuation stressors	–						
2. Life threat during	0.08	–					
3. Loss and disruption after	0.23^***^	0.22^**^	–				
4. Posttraumatic stress	0.42^***^	0.28^***^	0.40^***^	–			
5. Anxiety	0.26^***^	0.29^***^	0.29^***^	0.60^***^	–		
6. Depression	0.28^***^	0.26^***^	0.26^***^	0.74^***^	0.66^***^	–	
7. Somatic complaints	0.12	0.17^*^	0.14^*^	0.50^***^	0.46^***^	0.57^***^	–
8. Family income	−0.13^†^	−0.06	−0.19^**^	−0.18^**^	−0.05	−0.18^**^	−0.04

*p < 0.05, ^**^p < 0.01, ^***^p < 0.001.

† *p* = 0.05.

### 3.2. Testing the associations between evacuation stressors, hurricane exposure, psychological distress, and somatic complaints

**Figure 1:**
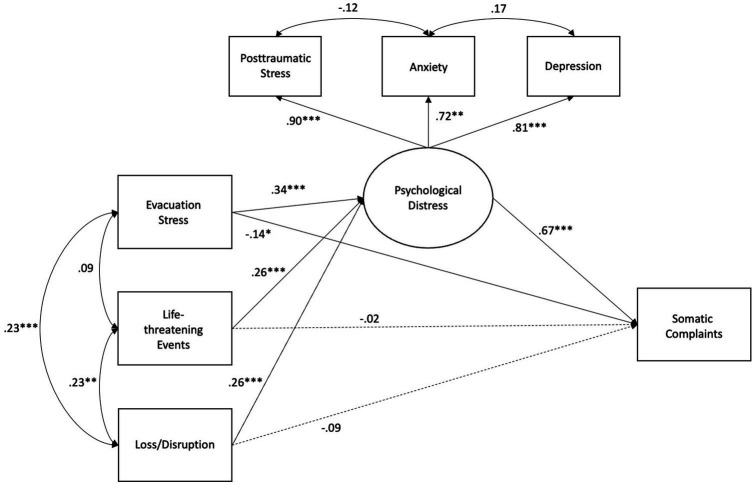
Path diagram for structural equation model, controlling for income and youth gender.

SEM evaluated whether evacuation stressors and hurricane exposure were directly associated with youth somatic complaints, and whether youth psychological distress served as a potential mediating pathway between evacuation stress, hurricane experiences, and youth somatic complaints. SEM revealed a good model fit (χ^2^ = 32.24, *p* = 0.003, CFI = 0.96, RMSEA = 0.08, SRMR = 0.04; see Figure 1 and Table 4).

**Table 4:** Path coefficients from the structural equation model.

	Unstandardized path coefficient	Standardized path coefficient	*SE*
**Direct effects**
*Youth psychological distress on*			
**Evacuation stressors**	0.10^***^	**0.34**	**0.02**
**Life-threatening events**	0.82^***^	**0.26**	**0.23**
**Loss/disruption**	0.77^***^	**0.26**	**0.20**
Income	−0.19	−0.09	0.13
Youth gender	−0.03	−0.01	0.31
*Youth somatic complaints on*			
**Evacuation stressors**	−0.04^*^	**−0.14**	**0.02**
Life-threatening events	−0.05	−0.02	0.21
Loss/disruption	−0.26	−0.09	0.18
Income	0.11	0.06	0.11
**Youth gender**	0.63^*^	**0.14**	**0.27**
**Youth psychological distress**	0.61^***^	**0.67**	**0.08**
*Income with*			
**Evacuation stressors**	**−1.36** ^†^	**−0.13**	**0.71**
Life-threatening events	−0.07	−0.07	**0.07**
**Loss/Disruption**	−0.21^**^	**−0.19**	**0.07**
*Youth anxiety with*			
Youth depression	0.96	0.17	0.78
Youth PTS	−1.84	−0.12	2.60
*Life-threatening events with*			
Evacuation stressors	0.60	0.09	0.48
**Loss/disruption**	0.15^**^	**0.23**	**0.05**
*Loss/disruption with*			
**Evacuation stressors**	1.65^**^	**0.23**	**0.49**
**Indirect effects**
*Youth somatic complaints on, via psychological distress*			
**Evacuation stressors**	0.06^***^	**0.22**	**0.01**
**Life-threatening events**	0.50^**^	**0.17**	**0.15**
**Loss and disruption**	0.47^**^	**0.17**	**0.14**

Significant effects are in bold text; *N* = 226. *p < 0.05, ^**^p < 0.01, ^***^p < 0.001.

† *p* < 0.10.

First, as expected, life-threatening hurricane experiences (*β* = 0.26; *p* < 0.001) and hurricane loss/disruption (*β* = 0.26; *p* < 0.001) were associated with greater symptoms of youth psychological distress; evacuation stressors also were uniquely associated with symptoms of youth psychological distress (*β* = 0.34; *p* < 0.001). Second, greater psychological distress was directly associated with more somatic complaints (*β* = 0.67; *p* < 0.001). Youths’ gender also was associated with somatic complaints, such that girls exhibited more somatic complaints than boys (*β* = 0.14, *p* = 0.02), although no significant findings were observed for family income, with all other variables included in the model. Finally, tests of indirect effects revealed that evacuation stressors (*β* = 0.22; *p* < 0.001), life-threatening events during the hurricane (*β* = 0.17, *p* < 0.01), and loss/ disruption after the hurricane (*β* = 0.17, *p* < 0.01) were all uniquely and indirectly associated with increased somatic complaints *via* youth psychological distress.

## 4. Discussion

Climate change is contributing to an increase in weather-related disasters and potentially traumatic weather events, which may not materialize, but which may require youth and families to evacuate their homes for safety. To our knowledge, this is the first study to examine the association between evacuation stressors experienced before a potential disaster and youths’ somatic complaints. Specifically, we found that evacuation stressors occurring *before* Hurricane Irma were uniquely associated with youths’ psychological distress (i.e., symptoms of PTS, anxiety, and depression), which in turn was significantly associated with youths’ somatic complaints 3 months after the storm. Below we discuss key findings from this study.

### 4.1. Disaster exposure and youths’ physical and mental health

Our findings regarding the associations between hurricane exposure (i.e., life-threatening events, loss and disruption) and youth’s psychological distress replicates earlier findings that directly link disaster exposure to posttraumatic stress and symptoms of anxiety and depression in youth (e.g., Bonanno et al., 2010; Furr et al., 2010; Lai et al., 2013). Although the level of hurricane exposure in this sample was not as high as in studies of highly destructive hurricanes (e.g., Andrew, Charley, or Katrina) (La Greca et al., 1996, 2010, 2013a,b; Hensley and Varela, 2008; Furr et al., 2010; Lai et al., 2013), the findings continue to document a “dose–response” relationship between youths’ hurricane exposure and their mental health outcomes. Further, although youths’ symptoms of psychological distress fell within the non-clinical range on average, a notable number of youth exceeded clinical cut-offs for depression and posttraumatic stress.

Importantly, the current study extends earlier findings on disaster exposure to a physical health outcome, specifically youths’ somatic complaints. Physical health is an understudied area of disasters’ impacts (La Greca et al., 2016). Our findings replicate those of Hensley and Varela (2008), who found an association between hurricane exposure and middle-school youths’ somatic complaints, and extend their findings to a much broader age range of youth (ages 7–17 years). Our findings are also compatible with a study of adult survivors of Hurricane Katrina (Arcaya et al., 2017), which found that disaster-related symptoms of PTS were associated with significantly higher odds of experiencing frequent migraines or headaches in the months after the hurricane.

Similar to Hensley and Varela (2008), we found that the somatic complaints of headaches and nausea/stomachaches were commonly reported, in addition to cold/flu symptoms, which can be an outcome of persistent stress (Cohen et al., 1991). Somatic symptoms, such as headaches and stomachaches, are associated with poorer health overall and contribute to impaired functioning in youth. For example, headaches are associated with impairments in youths’ daily functioning, school performance, and social activities (Kelly et al., 2018; Lyyra et al., 2018). Further, youth with chronic stomachaches (a.k.a. recurrent abdominal pain) have been found to develop other somatic symptoms and functional disability, such as work or school absence, several years later (e.g., Walker et al., 1995).

The impact of disasters on youths’ physical health continues to be an important area for future investigation. For example, it would be of interest to gather more details on the frequency and duration of headaches, stomachaches, and colds or flu that affect youth after disasters. Future studies also might expand the types of physical health outcomes that are assessed to include, for instance, lower respiratory infections, which also have been linked with stress and posttraumatic stress disorder in adults (e.g., Waszczuk et al., 2017). Finally, future work might expand the assessment of health outcomes to cover multiple timepoints.

### 4.2. Evacuation stressors and youths’ physical and mental health

Most significantly, our study findings indicate that stressors occurring *even before a disaster* have implications for youths’ physical and mental health, and that youth can be affected by stressors that occur before a disaster *regardless of whether the youth are directly exposed to the actual disaster.* Our findings likely reflect the considerable angst and uncertainty families experience before an impending disaster. For example, parents report concerns about family members’ safety and uncertainty about where, when, and how to evacuate, and indicate that the “worst part” of evacuating is “stress, worry, and uncertainty” (e.g., La Greca et al., 2019).

As such, greater attention to the pre-disaster period is warranted in future research, as even coping with the threat of a disaster may be sufficient to prompt psychological and physical health symptoms in youth. Notably, due in part to climate change, threats of disaster occur much more often than actual disaster exposure. With respect to hurricanes, for example, the 2020 U.S. Atlantic hurricane season included 30 named storms, yet only 11 storms made landfall (NOAA National Centers for Environmental Information, 2021).

Although the child disaster literature is largely limited to disasters that occurred, the psychological and physical health toll of storms (or other potential disasters, such as wildfires) that threatened but never materialized is an important area for further study. Such studies are challenging to conduct, due to the unpredictable nature of disasters. However, studies that conduct routine assessment or monitoring of parents’ and youths’ psychological and physical health in disaster prone areas, even before a disaster strikes, would enable us to better examine this issue. This type of research strategy would be especially useful in geographic regions that are chronically threatened by disasters, such as the coastal areas of Florida, Texas, and North Carolina, which are hurricane-prone (NOAA National Centers for Environmental Information, 2021), and California and the Northwestern U.S., which are key areas for wildfires (United States Environmental Protection Agency, 2023).

Further, in future research, the conceptualization of “disaster exposure” might be broadened to incorporate evacuation-related stressors that occur before a potentially traumatic event—rather than focusing solely on life-threat that occurs during an event or loss disruption that ensues afterwards. In the case of hurricanes, and other disasters such as floods or wildfires, pre-disaster stressors for families might include experiences such as deciding where and how to evacuate, and fears about personal and family safety (La Greca et al., 2019).

It will also be critical to examine the role of the media (and social media) in contributing to evacuation-related stress. Recent work with adults found that greater exposure to disaster-related media coverage before Hurricane Irma made landfall in FL was associated with poorer post-storm psychological adjustment (Thompson et al., 2019). Extending this line of research to the impact of media and social media on youth’s psychological and physical adjustment would be important and desirable.

### 4.3. Psychological distress as a potential mediator between disaster experiences and somatic complaints

The findings point to the role of psychological distress as a potential mediator between evacuation stressors, hurricane exposure, and youth’s somatic complaints. Although our findings were consistent with psychological distress as a mediator, they need to be confirmed in future studies using a prospective research design. Nevertheless, our findings are compatible with adult research linking disaster-related PTS with adults’ physical health problems, such as headaches (e.g., Arcaya et al., 2017) and lower respiratory infections (e.g., Waszczuk et al., 2017), and also with a study linking children’s hurricane-related symptoms of PTS with increased sedentary behavior (Lai et al., 2014).

In general, there is a large body of research linking symptoms of PTS with poor physical health after other kinds of traumatic events (e.g., military combat, sexual assault, and child abuse) but this association has not been well studied in the context of disasters (Jankowski, 2023). Moreover, as our findings suggest, symptoms of PTS may not be the only aspect of psychological distress that could contribute to youths’ physical health problems. PTS frequently co-occurs with symptoms of anxiety and depression in youth and adults (e.g., Bonanno et al., 2010; Lai et al., 2013; La Greca et al., 2013a,b; also see La Greca and Danzi, 2019), which was the case in the current study. In turn, symptoms of anxiety and depression have been linked with physical health problems, such as fatigue, stomachaches, and chronic health problems (e.g., Jones et al., 2017; Centers for Disease Control and Prevention, 2023).

Going forward, greater attention to disasters’ impact on youths’ health is warranted. Future disaster-based studies might incorporate multiple indicators of psychological distress, as we did here. In addition, more diverse measures of physical health are needed, especially ones that do not rely on self-report or parent-report, such as medical care utilization or physician diagnosis.

### 4.4. Implications of findings for disaster preparedness

The study findings regarding evacuation stressors have several important implications for disaster preparedness. First and foremost, disaster preparedness materials may need to be expanded to address the emotional and psychological needs of youth and families around the evacuation process. Although materials are available to help children or adults prepare for disasters (e.g., Ready.gov, 2023), the materials largely neglect mental health issues, such as how to manage and reduce the stress and anticipatory anxiety that parents and youth experience before a storm. In particular, it would be extremely valuable to expand existing materials to include “stress management” strategies for both youth and parents, such as limiting media exposure (Pfefferbaum et al., 2014) during the days leading up to a storm and encouraging family activities to promote relaxation (e.g., deep breathing, mindfulness, and exercise) (Newman and Motta, 2007; Cutright et al., 2019; e.g., La Greca and Sevin, 2023). Additionally, Family Disaster Plans could be expanded to include social–emotional aspects of family communication (e.g., how we will stay calm and speak to one another in a helpful manner, how often we will check in with each other about how we are feeling).

Second, in addition to expanding existing materials, concerted efforts to encourage uptake of relevant preparedness materials is needed. Ideally, this might be done in a systematic manner. For example, Family Disaster Plan instructions (Ready.gov, 2023) could be disseminated in medical and school settings, to coincide with the key times for potential disaster events (e.g., early Spring for tornadoes; summer for wildfires; late summer for hurricanes). Relevant materials could also be disseminated systematically at the beginning of the school year in areas that are vulnerable to climate-related extreme weather events. Such efforts would likely reduce the stress of “last minute” decision making.

We note that improving preparedness materials for parents and youth might not only prevent psychological distress and physical health problems, but also might help to reduce “over-evacuation.” In our study, for example, many families reported evacuating (76%) even though only half (50%) resided in evacuation zones. In fact, over a third (37%) of those not residing in mandatory zones evacuated. Over-evacuation creates problems for emergency management, as it potentially clogs highways and contributes to significant transportation issues and other evacuation stressors in the affected communities (e.g., Wolshon et al., 2005b; Archibald and McNeil, 2012). Over-evacuation also could prevent those who need to evacuate from being able to do so.

Finally, this study’s findings have important implications for disaster-related public health issues, such as eligibility for accessing mental and physical health services, as existing services may be targeted or limited to those with direct disaster exposure. However, even youth who evacuate to avoid the storm/disaster may exhibit psychological distress and physical health problems.

### 4.5. Study limitations

Several study limitations should be noted. First, although our evacuation-related measures were designed to capture objective experiences before a hurricane, data were collected at one time point after the hurricane. The findings suggest a potential mediating path between evacuation stressors and youth somatic complaints *via* psychological distress, consistent with the (scant) literature linking disaster-related stress with youths’ health problems and somatic complaints (e.g., Hensley and Varela, 2008). However, a multi-wave study would be valuable to replicate our findings and more directly evaluate the pathways by which pre-disaster evacuation stressors affect youths’ physical health. As noted previously, such studies are challenging to conduct, but would be especially valuable.

Second, we recruited parents via targeted Facebook ads; thus, our findings most directly generalize to youth whose parents are social media users. However, Facebook use is high among adults across diverse ethnic and socioeconomic backgrounds. For example, no differences have been observed among Black (70%), White (70%), and Hispanic adults (69%) in terms of their Facebook use; and Facebook use appears similar across diverse income levels (Perrin and Anderson, 2019). Further, adults who use Facebook may be an important group to study, as social media likely influences evacuation behaviors and could affect adults’ judgements of how bad a disaster may be.

Third, although we made efforts to obtain a diverse sample (e.g., advertising in Spanish, availability of the survey in Spanish), future research targeting multiple demographic groups is crucial for better understanding how evacuation stressors affect youth and yielding more generalizable findings. Notably, neither race nor ethnicity were related to our key study variables. However, low-income families reported more evacuation stressors, hurricane-related loss/disruption, and more symptoms of PTS and depression in their youth.

Fourth, although our findings were significant, the magnitude of the effects was modest. Most likely this is because youth did not experience high levels of disaster exposure overall. Even so, about 5% of the youth exceeded clinical cutoffs for PTS and over 15% exceeded cutoffs for depression. It is also notable that the effects of evacuation added to those associated with hurricane exposure. This finding is consistent with other research on children’s disaster responses where the effects of trauma exposure and other stressors appear to be cumulative (e.g., La Greca et al., 2010).

Finally, we relied on parents’ reports of their child’s functioning. Given that this was, to our knowledge, the first study to examine the impact of evacuation on child health, our recruitment strategy targeted parents, and it was not possible to also obtain youths’ reports. Generally, it is advisable to obtain reports directly from youth as parents may underreport youths’ internalizing symptoms (e.g., see Pfefferbaum et al., 2013). We note that our strategy of obtaining parent reports, if anything, may have underestimated the extent of youths’ problems. Thus, it is recommended that reports be obtained directly from youth in future research whenever feasible.

### 4.6. Summary

This study incorporated a broad conceptualization of disaster exposure that includes before-the-storm stressors and provides data on stressors’ associations with youths’ physical health, an understudied area. Findings demonstrate that evacuation stressors, as well as direct disaster exposure during and after an event, are uniquely associated with youth psychological distress, and in turn, with youth somatic problems.

## Data availability statement

The raw data supporting the conclusions of this article will be made available by the authors, without undue reservation.

## Ethics statement

The studies involving human participants were reviewed and approved by Human Subjects Research Office, University of Miami, Coral Gables, FL, United States. The patients/participants provided their written informed consent to participate in this study.

## Author contributions

AL had the primary responsibility for the design and implementation of the research project, obtaining IRB approvals, directing the study aims, analyses and write up of the study, and also was the lead writer in preparing the submitted manuscript. EB was primarily responsible for conducting the study analyses, preparing tables and a figure for the results, and also contributed to the writing and editing of the manuscript. KB was instrumental in the implementation of the research project, serving as a coordinator for data collection, preparation of online materials and tracking participants through the protocol, and also contributed to the write up and editing of the submitted manuscript. All authors contributed to the article and approved the submitted version.

## Funding

Funding for the initial project was supported by a small grant from the College of Arts and Sciences at the University of Miami, Coral Gables, FL. Open access publication fees were supported by several sources, including: the College of Arts and Sciences at the University of Miami; Department of Psychology Flipse Funds; and the Distinguished Professorship Funds for the AL.

## Conflict of interest

The authors declare that the research was conducted in the absence of any commercial or financial relationships that could be construed as a potential conflict of interest.

## Publisher’s note

All claims expressed in this article are solely those of the authors and do not necessarily represent those of their affiliated organizations, or those of the publisher, the editors and the reviewers. Any product that may be evaluated in this article, or claim that may be made by its manufacturer, is not guaranteed or endorsed by the publisher.
